# The New York Homœopathic Union

**Published:** 1897-12

**Authors:** E. D. Wilcox

**Affiliations:** Secretary


### NEW YORK HOMŒOPATHIC UNION.

The regular monthly meeting of the New York Homœopathic Union was held at 62 West Forty-ninth Street, New York, Thursday, November 18th, 1897, the President, Edmund Carleton, M. D., in the chair.

Present: Drs. Finch, Leverson, Spencer Carleton, Klein, Gould, and Wilcox.

The first part of the evening was devoted to consideration of clinical topics.

Dr. Leverson reported success in the treatment of two severe cases of small-pox by the use of Pickering's bath. This was given twice a day for two days, then daily for three days. In answer to questions, the Doctor stated that he also gave the remedy indicated homœopathically.

Dr. Finch had a case of small-pox some years ago which he reported to the Health Board and received permission to keep at home. It was turned over to a colleague, who was so situated that he could give proper attention. The patient died, and was buried by the Health officers in a place unknown to the family. A few days later the mother announced, with great lamentation, that her other son was sick, and she feared he would go the same way. The mother was reassured, and the son received a dose of *Variolinum* 200th, which soon cured him without much pitting.

Dr. Carleton had not seen a case of small-pox for a number of years. The last one was of frightful severity. The subject was a middle-aged gentleman, connected with the custom-house. He always believed that he had contracted the disease while superintending the unloading and weighing of a cargo of rags. At first he felt sick, but paid little attention to it until the hard nodules appeared. The sanitary inspector agreed to the diagnosis. The patient remained at home. He became a putrid, unrecognizable mass. The nurse was faithful to instructions, and not only gave the medicine but muffled the hands of the delirious man and kept him from himself. *Rhus-tox.* 200th, in water, a teaspoonful every two hours, cured so well that friends of the patient cried "fraud." Indications for the remedy not now remembered.

Dr. Finch—I had a family all down one after the other. First a daughter, who was unvaccinated. I treated her homœopathically, and she came out unscarred. The others were all vaccinated. The aunt next took it, and I cured her. Then the father, who had it lightly and kept to work. A few days after, in my absence, a brother was taken sick. An allopath was called in, who physicked him and he died.

Dr. Wilcox—I think if Dr. Leverson would try the Hahnemannian treatment he would not have to inflict such heroic measures on his patients, and he would find that the cure would be easier and as quick, if not more so. As Hahnemann states, we should treat in the gentlest, speediest, and most permanent manner, which we have all proven to be by *simillimum.*

The subject of fevers was then brought up.

Dr. Carleton—One case of remittent fever has come under my observation this autumn, in the person of a woman, forty years of age, who went South to take care of a very sick friend. While there, she was seized with what was called malarial fever, and received for it enormous doses of Quinine and a good deal of Calomel. As soon as she could be dressed and put into a sleeping-car, she came North.

Temperature ranged from 99.6 to 103, requiring over two days to run up and down that scale. Aside from an almost constant feeling that she wanted more clothing, a little chilly perhaps, and insomnia, it seemed about impossible to get symptoms. However, while she ran up and down the scale twice, I did manage to get the following: Large, loose stool in a hurry, 6 A. M.; cramps in calves at night; felt worse after bathing. It was evident that a peculiar mental state existed; but I never succeeded in analyzing it.

*Sulphur* 200th (Jenichen), in water, every two hours until relieved and then stop, brought improvement in four days. One week after that she had normal temperature, but felt very weak. Convalescence was rapid and uninterrupted. So far there has been no return of any trouble. I expect a reckoning next spring with Georgia Physic & Co.

Dr. Finch—Did this case begin as remittent or intermittent?

Dr. Carleton—I am unable to say. She was in the South, and the old-school gentleman began his heavy dosing at once. That destroyed the type.

By previous arrangement, the next subject considered was, "*Can Susceptibility to Disease be Cured?*"

Dr. Carleton—We have been asked to consider that question this evening.

My answer is in the affirmative. The testimony of provers is that by proving drugs as required by Hahnemann they have made themselves more robust and less susceptible to disease. On this point we have the testimony of Hahnemann himself in *The Organon*, Section 141, with note. It reads thus:

"But of all the pure experiments relative to the changes which simple medicines produce, and the morbid symptoms they excite in healthy persons, those are always best which a physician (enjoying a good state of health, free from prejudice, and able to analyze his sensations) makes on his own person, observing, at the same time, the precautions that have just been described. A thing is never more certain than when it has been tried on ourselves.

"(Note.)—The experiments that are made on our own persons have one advantage above all others. In the first place, they furnish a conviction of this great truth, that the curative virtues of medicine depend solely upon the power they possess of creating changes in the physical economy of man. In the second place, they teach us to understand our own sensations, mind, and disposition, which is the source of all true wisdom, and exercise our powers of observation, an indispensable talent in a physician. All our observations on others are by no means so interesting as those made on ourselves. In all the observations made on other individuals, it is certainly to be feared that the person making trial of the remedy may not exactly experience that which he says, or will not express in a proper manner that which he feels.

"The physician must always remain in doubt, or at least partly so, whether he is deceived or not. This obstacle to a knowledge of the truth, which cannot be entirely obviated in a search after the morbid symptoms excited in another person by the action of the remedy, does not exist where the trial is made on our own persons. The individual who undergoes the experiment knows precisely what he feels, and every fresh attempt that he makes is an additional motive for him to extend his researches still farther, by directing them towards other remedies. It renders him more expert in pursuing farther trials, while, at the same time, his zeal is redoubled, because he thereby acquires a true knowledge of the resources of the art, which can be considerably increased. Do not let him suppose, on the other hand, that the slight inconveniences which he subjects himself to in trying the medicines on his own person can be detrimental to his health.

"On the contrary, experience has shown us that they only render the body more apt to repel all natural and artificial morbific causes, and harden it against their influence. The same experience also teaches that thereby the health becomes more firm, and the body more robust."

Another way in which susceptibility to disease can be overcome is by homœopathic medication. When symptoms appear, let them be met by the similar remedy. Each recurrence will thereby become less frequent, less severe, and more quickly stopped. In other words, susceptibility will be lessened. All have seen cases of hay fever take that course, and other cases of intermittent fever, each cured with a single dose of medicine, so that they all could with impunity continue to reside in the malarious districts that had made them sick, instead of being obliged to flee the country or be ill, as before when under allopathic control.

Looking about us we see multitudes of people who have been made immune against disease, either by occupation or accident. Miners of Mercury are proof against syphilis, and those of Copper against cholera. Our old-school neighbors declare that erysipelas cannot be followed by cancer. One attack of whooping-cough, or scarlet fever, measles, mumps, or other zymotic disease usually fortifies against another attack. Proofs might be multiplied, but the foregoing will suffice to establish the truth of our position.

Dr. Finch—That is *strictly true* about immunity from malarial poison.

Dr. Wilcox—A lady came to me saying she felt bad all over and wanted me to try to help her. She said she had indigestion, trouble with her liver, her chest, and she knew not what. In taking her case she spoke of not breathing well, but dismissed it with, "But that I have always had because the bones of my nose are too close together, and I must breathe through my mouth. That makes me get a sore throat every time I go into the wind. My old doctor told me to have an operation, but I will not. I had rather have colds all the time."

When I had all the symptoms I told her I would send the medicine. After studying the case I prescribed *Kali-carb.* CM. Two weeks later she came to me feeling very much better in every way, and said, "Doctor, I can breathe through my nose even in this cold wind, and I was out several days without catching cold."

Dr. Finch—In many cases susceptible to catching cold in cold air I find that Sulph., Dulc., Nux-vom., or Rhus will clear up the sensitiveness, by removing the catarrhal diathesis.

One thing we must remember, and that is, that if a patient has any dyscrasia it will influence every sickness, but if we take it into consideration with every prescription it will gradually clear up and the susceptibility to disease be gone.

Dr. Leverson—I find that age has a great deal to do with rendering success in that line difficult.

Dr. Carleton—Hahnemann proved many of the remedies on himself during a great number of years and found no difference.

Dr. Finch—In reference to provers they are told to report any symptoms they may be subject to beforehand, and if those symptoms are influenced by the drug they are called clinical. I will tell you of an unexpected proving of Glonoine made by me: Mr. Smith was making Glonoine and had some undiluted Nitro-glycerine. Dr. Maars came in. He was one of the original seven provers with Gram. He said: "Give me some for a proving." Mr. Smith took a small glass bulb, immersed it, shook off most of the liquid, and touched the Doctor's tongue. In five minutes the pulse went up and his face became white. I did the same. I felt my hair stand up and a choking in the neck. Blood rushed to my head, and I felt queer and weak. Nausea in the throat that remained months. Sensation as if top of head would fly off. Every tooth ached and wherever there were fillings they throbbed. My hair remained up for some time.

Dr. Wilcox—The best answer to the question of the evening is when our patients tell us that they do not have so much sickness under homœopathic treatment, and that they do not take sick so easily.

Adjourned.

E. D. Wilcox,
*Secretary.*

